# Supplementary material for: Auditing the impact of social media’s policy shift on anti-vaccine discourse: A large language model-driven empirical study
Source: PLoS One. 2026 Apr 29;21(4):e0346568. doi: 10.1371/journal.pone.0346568 (PMC13127958; doi:10.1371/journal.pone.0346568)
Supplement: Attachment — Submitted filename: response_to_reviewers_1227.docx [file pone.0346568.s001.docx]

# Response to Reviewers

**Manuscript ID:** PONE-D-25-17346
**Title:** Auditing the Impact of Social Media’s Policy Shift on Anti-Vaccine Discourse: A Large Language Model-Driven Empirical Study
**Journal:** PLOS ONE

We thank the Academic Editor and the three reviewers for their constructive feedback. We have carefully addressed each comment and made substantial revisions to the manuscript. Below is our point-by-point response.

## Response to Academic Editor

*“For acceptance, please validate the LLM labels with a small human double-coding and report precision/recall/F1; show counts and proportions with odds ratios and 95% CIs; add simple robustness checks for outliers, retweets/duplicates, and bots; clarify dataset timing, filters, and category scheme; share data/code per PLOS policy; soften causal language; update figures to include proportions with accessible colors; fix reference formatting and typos. Nice to have: tighten the research gap and early definitions, and expand the discussion to acknowledge alternatives and limits.”*

**Response:** We have addressed all required revisions and suggested improvements.

For LLM validation, we added external validation using the CAVES benchmark (F1=0.883) and cross-annotator agreement analysis (κ=0.71-0.86) in Section 3.2. We report total sample sizes (N=13,458) and class distribution statistics in Section 4.1, with sensitivity analyses excluding highly retweeted content in Section 4.2. The Banda et al. dataset timing and filtering process are clarified in Section 3.1, and a data availability statement has been added per PLOS policy. Throughout the manuscript, we removed “natural experiment” framing and softened causal claims. All figures now display percentages with a colorblind-friendly palette, and reference formatting issues have been corrected.

We have tightened the research gap statement and added early definitions of key constructs (amplification, thematic composition, stance consistency) in the Introduction. We have also expanded the Discussion and Limitations sections to acknowledge alternative explanations and the limits of causal inference in our observational design.

## Response to Journal Requirements

### Requirement 1: Style Requirements

*“Please ensure that your manuscript meets PLOS ONE’s style requirements, including those for file naming.”*

**Response:** We have reviewed and ensured that the manuscript conforms to PLOS ONE’s style requirements, including file naming conventions, formatting, and structure.

### Requirement 2: Data Availability

*“We note that you have indicated that there are restrictions to data sharing for this study… Please address the following prompts regarding ethical or legal restrictions on sharing data.”*

**Response:** Our study uses tweet IDs from the publicly available Banda et al. COVID-19 Twitter dataset. Due to Twitter/X’s Terms of Service, we cannot directly share tweet text content. However, we will share: (a) the complete list of tweet IDs used in our analysis, (b) our annotation results (stance labels, theme labels) linked to tweet IDs, and (c) all analysis code. Researchers can rehydrate the tweets using the provided IDs, subject to Twitter/X’s data access policies. We have updated the Data Availability statement accordingly.

### Requirement 3: Figure References

*“Please ensure that you refer to Figures 4 and 5 in your text as, if accepted, production will need this reference to link the reader to the figure.”*

**Response:** We have verified that Figures 4 and 5 are properly referenced in the manuscript text.

### Requirement 4: Table References

*“Please ensure that you refer to Tables 1 and 4 in your text as, if accepted, production will need this reference to link the reader to the table.”*

**Response:** We have verified that Tables 1 and 4 are properly referenced in the manuscript text.

### Requirement 5: Reviewer-Recommended Citations

*“If the reviewer comments include a recommendation to cite specific previously published works, please review and evaluate these publications to determine whether they are relevant and should be cited.”*

**Response:** Reviewer #3 recommended four publications regarding echo chamber literature (Barberá 2020; Guess et al. 2018; Bail et al. 2018; Törnberg 2022). Upon review, we found these works highly relevant and they informed our decision to remove the echo chamber framing from the manuscript (see Comment 3.3). While we did not cite these works directly (as we removed the echo chamber discussion rather than revising it), we acknowledge their importance in shaping our revision.

## Response to Reviewer #1

### Comment 1.1: LLM Validation

*“The use of GPT-4o for tweet classification and thematic labeling should be better justified. In section 3.2, it is simply stated that ‘To verify annotation quality, we randomly sampled 200 labeled tweets for manual review by the research team and found that the LLM performed very well.’ This point must be justified in a more rigorous (statistical) manner.”*

**Response:** We have substantially strengthened our LLM validation through two approaches: 1. **External benchmark validation:** We tested our stance classification prompt on the CAVES dataset (Poddar et al., 2022), a COVID-19 vaccine stance benchmark developed by the same research team that established the thematic taxonomy we adopted. CAVES contains 1,977 expert-labeled tweets with stance annotations. Our classifier achieved F1 = 0.883 for anti-vaccine stance identification (Precision = 0.951, Recall = 0.824), demonstrating strong generalization beyond our study dataset. 2. **Cross-annotator agreement:** We randomly sampled 500 tweets from our dataset and obtained independent annotations from two additional annotators using identical classification criteria. Inter-annotator agreement measured by Cohen’s κ ranged from 0.71 to 0.79 for stance classification and 0.72 to 0.86 for theme classification, indicating substantial agreement.

**Changes:** See revised Section 3.2 (Annotation Validation).

### Comment 1.2: Sample Size and Class Balance

*“On top of that, the manuscript does not explicitly state the total number of tweets analyzed after filtering and annotation; if the classes are balanced and so on…”*

**Response:** We have added detailed sample statistics at the beginning of the Results section. The final dataset comprises N=13,458 tweets (5,896 pre-policy, 7,562 post-policy), distributed across original tweets (18.2%), retweets (74.3%), and quote tweets (7.5%). Regarding vaccine stance, tweets were classified as anti-vaccine (49.6%), neutral (29.3%), pro-vaccine (18.1%), or no stance (2.9%), yielding a near-balanced classification between anti-vaccine and non-anti-vaccine content.

**Changes:** See revised Section 4.1 (Results).

### Comment 1.3: Self-Contained Explanations

*“The paper should be made more self-contained to make it easier to read, especially when dealing with importants points, eg: page 3: ‘assigned the identified antivaccine tweets into eight thematic categories [8]’; page 6: ‘we applied an additional keyword filtering step inspired by [42]’; page 7: ‘into eight thematic categories using GPT-4o, following the framework in [8]’”*

**Response:** We appreciate this feedback. Our original manuscript already included tables with definitions (Table 2 for eight anti-vaccine themes, Table 1 for keyword examples) and inline keyword examples (“mRNA vaccine”, “booster shot”, “Pfizer”, “Moderna”). However, we acknowledge that the reviewer may have found it inconvenient to locate these details. We have improved the manuscript by: - Adding an explicit table reference sentence: “Table 2 presents the eight anti-vaccine theme categories with definitions and examples” - Expanding the methodological explanation of how the eight-category taxonomy was derived (LDA topic discovery, human expert consolidation, Labelled-LDA refinement)

**Changes:** See revised Section 3.2 (Anti-Vaccine Theme Categorization).

### Comment 1.4: Dataset Timing Mismatch

*“According to section 3.1, the COVID19 dataset is taken from ref. [41] that was published in 2021. But the dataset of your study goes over the period Nov 16-30, 2022. How do you explain this mismatch?”*

**Response:** We apologize for the confusion. The Banda et al. dataset is a continuously updated repository that has been collecting COVID-19 tweets since January 2020 and continues to be updated. While the methodology paper was published in 2021, the data collection is ongoing. We have clarified this in Section 3.1.

**Changes:** See revised Section 3.1.

### Comment 1.5: Figures - Percentages Instead of Raw Counts

*“All the figures show raw data (Tweet count). It would be more informative to have it in percentage. Indeed, if the number of anti-vax Tweets increases together with the number of pro-vax, what really matters, is the relative proportion of the two.”*

**Response:** We agree. We have revised all figures to show proportions/percentages rather than raw counts, enabling better comparison of relative changes between periods.

**Changes:** See revised Figures 1-5.

### Comment 1.6: Reference Formatting

*“There are many bugs in the references that should be corrected: [4] and [28]: the name of the journal does not appear; [10], [13], [14]: question marks appearing in the references. This shows that the bibliographic research was botched. Furthermore, there are numerous X's studies about the COVID-19 into Plos One, and no one is quoted.”*

**Response:** We have corrected all reference formatting issues. Additionally, following the reviewer's suggestion, we added five recent PLOS ONE studies on COVID-19 vaccine discourse and misinformation to better situate our work within this journal's literature.

**Changes:** See revised References section.

### Comment 1.7: Tone Down Conclusions

*“The major flaw of the study, as explained in section 5.4, is the short time Windows, covering only seven days before and after the policy termination. There is a dissonance with the conclusions of section 5.3 (‘This study provides important insights into content moderation policies on social media platforms and their broader implications for public health communication’; ‘X’s misinformation policy may have played a crucial role in mitigating the spread of anti-vaccine discourse’; ‘anti-vaccine narratives gained greater visibility’) that should be re-written to take into account the small period of the study. In other word, the conclusion should be way more modest, in order to be in phase with the study.”*

**Response:** We agree. We have revised the conclusions to better reflect the limited 7-day observation window and removed overgeneralized claims. The specific phrases cited by the reviewer have been toned down or removed.

**Changes:** See revised Section 6 (Conclusion).

## Response to Reviewer #2

### Comment 2.1: Research Gap and Definitions

*“The Introduction is well-written and clearly structured; however, I suggest it could be improved by explicitly stating the research gap, defining key constructs like stance consistency and amplification earlier, and briefly framing the ‘marketplace of ideas’ concept to strengthen its theoretical grounding.”*

**Response:** We appreciate the positive feedback and have implemented the key suggestions: - **Research gap:** Added explicit statement: “While extensive research has examined the *implementation* of content moderation policies and their effectiveness in reducing misinformation spread, far less is known about the consequences of *removing* such policies once established.” - **Key construct definitions:** Added definitions for our three analytical dimensions: “(1) *amplification*, the degree to which anti-vaccine content spreads through retweets; (2) *thematic composition*, the specific narratives expressed in anti-vaccine discourse; and (3) *stance consistency*, whether users quoting vaccine-related tweets express agreement or disagreement with the original content.”

**Changes:** See revised Section 1 (Introduction).

### Comment 2.2: Related Work

*“In the Related Work section, the authors mention that few studies explore the removal of moderation policies; however, I believe this distinction could be made sharper by more explicitly contrasting it with the broader literature on policy implementation and clarifying how the current study fills that specific gap in relation to the stated research questions.”*

**Response:** We have revised the Related Work section to sharpen the distinction between our study and prior literature. Specifically, we removed the echo chamber framing (per Reviewer #3’s suggestion) and added recent PLOS ONE studies on COVID-19 vaccine discourse (Broniatowski et al. 2022; Hwang et al. 2022; Lanier et al. 2022; Hobbs et al. 2024; Mønsted & Lehmann 2022) to better situate our work within the current literature on platform governance and vaccine misinformation.

**Changes:** See revised Section 2 (Related Work).

### Comment 2.3: LLM Annotation Details

*“In the Methodology section, the authors briefly mention that 200 annotated tweets were manually reviewed to verify GPT-4o’s performance, but I suggest including more detail, such as accuracy rates, confidence thresholds, or inter-rater agreement metrics, to improve transparency and support the robustness of the annotation process.”*

**Response:** This concern was also raised by Reviewers #1 and #3. We have substantially strengthened our validation with two approaches: 1. **External benchmark validation:** We tested our classifier on the CAVES dataset (Poddar et al., 2022), achieving F1 = 0.883 for anti-vaccine stance identification (Precision = 0.951, Recall = 0.824). 2. **Inter-annotator agreement:** We randomly sampled 500 tweets and obtained independent annotations from two additional annotators. Cohen’s κ ranged from 0.71 to 0.79 for stance and 0.72 to 0.86 for themes, indicating substantial agreement.

**Changes:** See revised Section 3.2 (Annotation Validation).

### Comment 2.4: Duplicate Content and Bot Activity

*“While the inclusion of retweets is justified for amplification analysis, I suggest briefly discussing how potential duplicate content or bot-driven activity was handled or could have influenced the findings.”*

**Response:** We conducted sensitivity analyses to address concerns about content amplification. By excluding tweets with ≥50 retweets (30 original tweets and 5,407 associated retweets, 40.2% of data), we found that the retweet effect was attenuated while the original tweet effect increased (OR: 1.41→1.57), suggesting the policy change was associated with new content creation rather than merely viral amplification. Regarding bot activity, the dataset lacks user-level metadata for bot detection; we acknowledge this as a limitation.

**Changes:** See revised Section 4.2 (Sensitivity Analysis) and Section 5.5 (Limitations and Future Directions).

### Comment 2.5: Control Variables

*“The logistic regression models are appropriate, but I suggest briefly noting whether any control variables were considered to account for confounding factors, or clarifying the rationale for using a single binary predictor.”*

**Response:** Our design incorporates confounding control through temporal matching rather than regression-based adjustment. The 14-day window (7 days pre/post) ensures day-of-week matching (Monday vs. Monday, etc.), controlling for weekly cyclical patterns in posting behavior. This short duration also minimizes threats from secular trends, as user composition and platform characteristics are unlikely to change systematically within two weeks. This approach resembles matched pre-post designs used in epidemiological studies. We have clarified this rationale in Section 5.5, stating: “our regression models use a single binary predictor (pre/post policy) without additional control variables. This parsimonious specification reflects our short 14-day observation window, during which user composition and platform characteristics are unlikely to change systematically.”

**Changes:** See revised Section 5.5 (Limitations and Future Directions).

### Comment 2.6: Non-Significant Results

*“In the Results section, while the authors report that quoted tweets did not show a significant change post-policy, I suggest including the actual odds ratio and confidence interval to allow readers to interpret the strength and direction of the effect, even if not statistically significant.”*

**Response:** We appreciate the suggestion. In fact, our original manuscript already reported the OR and CI for quoted tweets: “quoted tweets did not demonstrate a statistically significant change (OR = 0.98, 95% CI: 0.77–1.26, p > 0.05).” We have verified that all comparisons throughout the Results section consistently report OR, 95% CI, and p-values, including non-significant results.

**Changes:** No change needed; statistics were already reported in the original submission.

### Comment 2.7: Raw Tweet Counts

*“To help contextualize the reported odds ratios, I suggest including raw tweet counts before and after the policy shift, particularly for anti-vaccine tweets in each tweet type (original, quote, retweet).”*

**Response:** We have added a new paragraph at the beginning of Section 4.1 reporting detailed sample statistics: “The final annotated dataset comprised 13,458 tweets: 5,896 from the pre-policy period (November 16–22) and 7,562 from the post-policy period (November 24–30). By tweet type, the dataset included 2,444 original tweets (18.2%), 10,001 retweets (74.3%), and 1,013 quote tweets (7.5%). Regarding vaccine stance, 6,674 tweets (49.6%) were classified as anti-vaccine, 3,949 (29.3%) as neutral, 2,442 (18.1%) as pro-vaccine, and 393 (2.9%) had no vaccine stance mentioned.”

**Changes:** See revised Section 4.1 (Results), first paragraph.

### Comment 2.8: Confounding Factors and Causal Limitations

*“However, I suggest expanding the discussion by addressing potential confounding factors beyond the policy change, such as algorithm shifts or concurrent news events, and by adding practical recommendations for platform moderation or public health communication strategies. It may also strengthen the paper to acknowledge the limitations of causal inference in a natural experiment design and the potential biases introduced by LLM-based classification.”*

**Response:** We have expanded the Limitations section to explicitly address these concerns. Specifically, we added: “our pre-post observational design precludes causal inference, as the period surrounding the policy termination was characterized by multiple concurrent events. Our analysis captures changes associated with the *announcement* of policy termination rather than changes in *enforcement* practices… potential confounders such as concurrent news events or algorithm changes cannot be adequately captured through tweet-level covariates.”

We also note that our design employs temporal matching (7 days pre/post with day-of-week alignment) to control for cyclical patterns, and stratified analyses by tweet type partially address confounding. However, we acknowledge that causal inference is not possible with our observational approach.

**Changes:** See revised Section 5.5 (Limitations and Future Directions).

### Comment 2.9: Conclusion and Future Directions

*“In the Conclusion section, the authors provide a concise summary of their study, but I suggest enhancing it by including a brief reflection on limitations and offering directions for future research, such as analyzing longer-term impacts, cross-platform effects, or applying alternative annotation methods.”*

**Response:** We have enhanced both the Limitations and Conclusion sections. Section 5.5 (Limitations and Future Directions) now explicitly discusses: (1) the short observation window, (2) the inability to infer causality from pre-post designs, (3) potential confounders, and (4) the lack of user-level analysis. The revised Conclusion (Section 6) notes that “our pre-post observational design and the presence of concurrent events during the study period preclude definitive causal conclusions” and suggests that future work could extend the observation window, incorporate user-level metadata for bot detection, and examine policy changes across multiple platforms.

**Changes:** See revised Section 5.5 (Limitations and Future Directions) and Section 6 (Conclusion).

## Response to Reviewer #3

### Comment 3.1: LLM Validation (Critical)

*“The most important issue with the paper is that the GPT-4o models (stance detection, topic detection, stance consistency) have not been validated. Not only should you report performance metrics like F1, precision, recall, etc., the members of the research team should independently annotate tweets for stance/topic, and the annotations should have sufficient inter-annotator agreement.”*

**Response:** We fully agree that this is a critical issue—one that was also raised by Reviewers #1 and #2. We have implemented a two-pronged validation approach: 1. **External benchmark validation:** We tested our classifier on the CAVES dataset (Poddar et al., 2022), which was developed by the same research team that established the thematic taxonomy we adopted. CAVES contains 1,977 expert-labeled tweets. Our classifier achieved F1 = 0.883 for anti-vaccine stance identification (Precision = 0.951, Recall = 0.824), demonstrating strong generalization beyond our study dataset. 2. **Cross-annotator agreement:** We randomly sampled 500 tweets from our dataset and obtained independent annotations from two additional annotators using identical classification criteria. Cohen’s κ ranged from 0.71 to 0.79 for stance and 0.72 to 0.86 for themes, indicating substantial agreement per Landis & Koch (1977) benchmarks.

**Changes:** See revised Section 3.2 (Annotation Validation).

### Comment 3.2: Causal Claims (Critical)

*“The causal claims made in the paper should be toned down. I think it’s a bit strong to say this is a natural experiment, since there was so much going on at this time that it’s easy to imagine some other factor explaining the rise in anti-vaccine content. Furthermore, it’s not clear what aspects of the moderation policy would have contributed to the increase in anti-vaccine content (is it just the announcement of the policy change that led people to post more content, or is it how the policy was actually enforced? Or is it just that so many people were laid off, there were fewer people to remove harmful posts?). In actuality, you are measuring the effect of the announcement of the removal of a moderation policy rather than the enforcement of the policy itself. I wouldn’t call this a ‘natural experiment,’ and I would remove all statements making causal claims. I would also mention as a limitation that you can’t infer causality.”*

**Response:** We concur with this assessment and have made extensive revisions throughout the manuscript. We replaced all instances of “natural experiment” with “pre-post observational design” and systematically softened causal language—for example, changing “impact of policy termination on anti-vaccine discourse” to “association between policy termination and changes in anti-vaccine discourse,” replacing “led to” with “was associated with,” and softening “demonstrates” to “suggests.”

Following the reviewer’s insight that we are measuring effects of the announcement rather than enforcement, we added explicit acknowledgment in the Limitations section: “Our analysis captures changes associated with the *announcement* of policy termination rather than changes in *enforcement* practices.” We also expanded this section to address potential confounders, noting that “concurrent news events or algorithm changes cannot be adequately captured through tweet-level covariates.” Finally, we added a clear statement in the Conclusion acknowledging that “our pre-post observational design and the presence of concurrent events during the study period preclude definitive causal conclusions.”

**Changes:** See revised Introduction, Methods, Results, Discussion, Conclusion, and Limitations sections.

### Comment 3.3: Echo Chamber Discussion

*“I would say prior literature largely disagrees with the way ‘echo chambers’ are described in this paper. You should consider Barberá (2020), which describes how people are more likely to be exposed to cross-cutting content online than offline, and reviews the literature on the role algorithms play in such interactions. Guess et al. (2018) provide another convincing review. You also imply in the discussion that cross-cutting interactions would decrease polarization, but Bail et al. (2018) find the opposite effect, and Törnberg (2022) provides a potential explanation for why this might be. Please review this literature before submitting your revision, and consider removing or significantly altering your discussion about echo chambers.”*

**Response:** We thank the reviewer for highlighting this literature. Upon reflection, we agree that our original echo chamber framing was overly simplistic and not well-supported by the nuanced findings in the literature. Rather than attempting to revise this discussion with the caveats it would require, we have chosen to remove the echo chamber framing entirely. Specifically, we replaced “algorithmically driven echo chambers” with “algorithmic amplification and community clustering” in the Related Work section, removed references to “echo chamber dynamics” in the Discussion, and deleted the extended comparison between Musk’s “digital town square” vision and echo chamber effects. Our revised discussion now focuses more narrowly on the empirical findings—increased stance consistency in quote tweets—without making broader claims about echo chamber formation.

**Changes:** See revised Related Work (Section 2) and Discussion (Section 5).

### Comment 3.4: Viral Tweet Analysis / Outliers

*“I am also curious how much of the increase in anti-vaccine content you observe can be explained by the big spike you see on day one, and how much of the increase in retweets can be attributed to the same tweets being retweeted a bunch of times. Is it possible that there was just a really viral tweet on the day the policy was enacted that results in the statistically significant changes? If you remove the outliers, will you still see a statistically significant result?”*

**Response:** This is an important concern that we have addressed through both our original study design and newly added sensitivity analyses.

Regarding the “day one” spike: our original regression models already excluded the announcement date itself (November 23), comparing only November 16–22 (pre-policy) with November 24–30 (post-policy). This design choice means that any spike occurring specifically on the announcement date does not directly influence our statistical results.

To further address the reviewer’s concern about whether results were driven by a small number of highly retweeted posts, we conducted additional sensitivity analyses. We excluded tweets with ≥50 retweets, which removed 30 original tweets and their 5,407 associated retweets (40.2% of the data). When we re-ran the regression on this filtered dataset, the retweet-specific effect was substantially attenuated and no longer statistically significant. However, the effect for original tweets increased (OR: 1.41→1.57), suggesting that the policy change was associated with the creation of new anti-vaccine content rather than merely amplifying a few viral posts.

We also added a new figure showing the distribution of the top 20 most retweeted tweets. Notably, despite having shorter observation windows for accumulating retweets (0–6 days vs. 8–14 days for pre-policy tweets), post-policy content comprised 50% of these highly retweeted tweets—including a November 23 post promoting the “Died Suddenly” documentary that directly appealed to Elon Musk not to censor it.

**Changes:** See revised Section 4.2 (Sensitivity Analysis) and new Figure showing distribution of highly retweeted content.

### Comment 3.5: Figure Improvements

*“I would also rework the plots. Please use a colorblind-friendly palette (i.e., not red/green). I also find the stacked bar chart difficult to read, since the axis for certain groups doesn’t start at zero, and the same stances for pre/post might not start at the same number. I would instead do three subplots for each tweet type, and have side-by-side bars for each stance showing the pre/post change.”*

**Response:** We have redesigned the figures to address the reviewer’s concerns. To avoid red/green color issues, we replaced the red dashed line (policy change marker) with a black dashed line throughout. For Figure 3 (pre/post comparison), we replaced the stacked bar chart with a grouped bar chart using solid bars for pre-policy and hatched bars for post-policy, enabling direct comparison at each stance category. All y-axes now display percentages rather than raw counts, with percentage labels added above bars to facilitate interpretation.

We note that Figures 1, 2, and 5 remain as stacked area plots because they display temporal trends where stacking effectively shows compositional changes over time. Figure 2 already included three subplots for tweet types (Original/RT/QT) in the original submission.

**Changes:** See revised Figures 1-5.

### Comment 3.6: Typo in Tables

*“There’s a typo in tables 6/7 (pro-policy instead of post-policy)”*

**Response:** This has been corrected. Thank you.

**Changes:** See revised Tables 6-7.

## Summary of Major Changes

| Section | Changes Made |
| --- | --- |
| Abstract | Softened causal language; added key metrics |
| Introduction | Removed “natural experiment”; clarified research gap; defined key terms (amplification, thematic composition, stance consistency) |
| Related Work | Removed echo chamber framing; added recent PLOS ONE studies (Broniatowski, Hwang, Lanier, Hobbs, Mønsted & Lehmann) |
| Methods 3.1 | Clarified dataset timing; explained filtering steps; justified retweet inclusion |
| Methods 3.2 | Added LLM validation (CAVES benchmark F1=0.883, cross-annotator κ=0.71-0.86) |
| Results 4.1 | Added sample sizes, class distribution, raw counts |
| Results 4.2 | Added sensitivity analysis (excluding highly retweeted content); added Figure showing top 20 retweeted tweets |
| Discussion | Removed echo chamber framing; added new Methodological Contributions subsection; refined comparison with prior literature |
| Limitations and Future Directions | Added causal inference caveat; justified single binary predictor design; acknowledged bot detection limitation; added future research directions |
| Conclusion | Toned down claims to match 7-day observation window; added causal limitation acknowledgment |
| Figures | Percentages, colorblind-friendly palette (black dashed line), grouped bars for Figure 3 |
| References | Fixed formatting issues; added PLOS ONE COVID-19 studies |
| Data Availability | Added statement per PLOS policy |

We believe these revisions substantially strengthen the manuscript and address all reviewer concerns. We are grateful for the opportunity to improve our work.

Sincerely,
The Authors
